# Supplementary material for: The Pattern of RNA Editing Changes in Pleural Mesothelioma upon Epithelial-Mesenchymal Transition
Source: Int J Mol Sci. 2023 Feb 2;24(3):2874. doi: 10.3390/ijms24032874 (PMC9917482; doi:10.3390/ijms24032874)
Supplement: Supplementary file 1 [file ijms-24-02874-s001.zip › Figure S1.pdf]

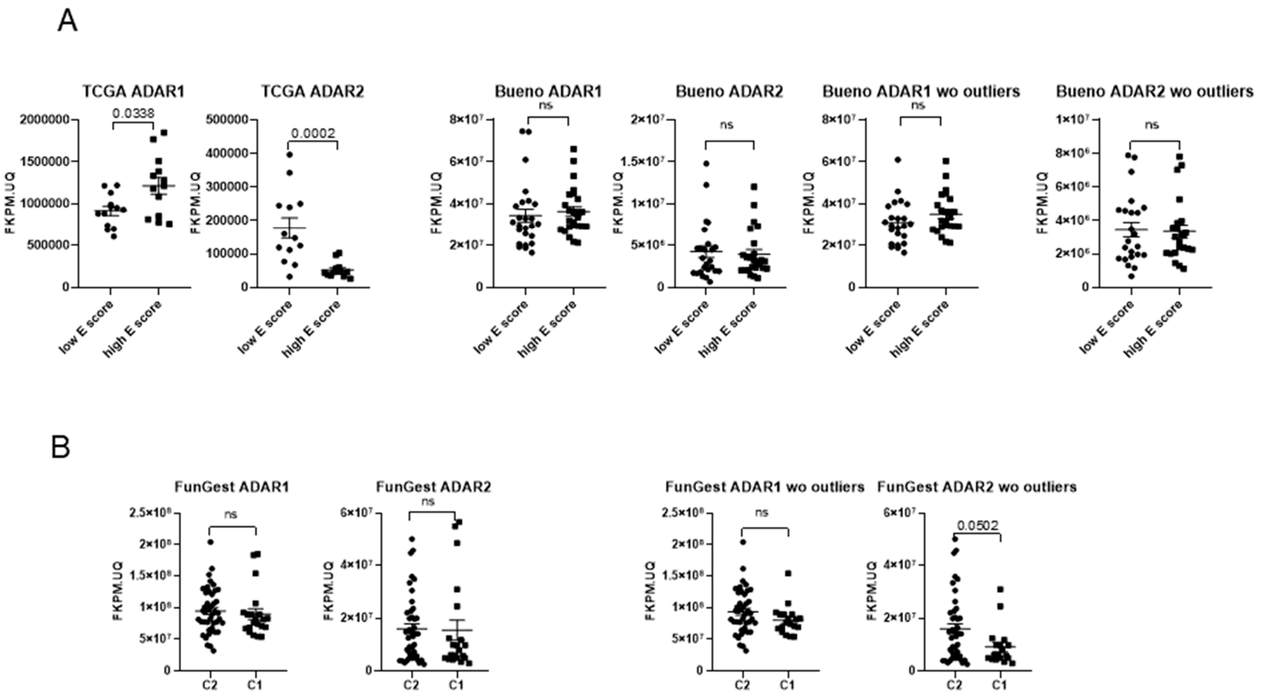

Figure S1. Differential expression of ADAR1 and ADAR2 in low vs high E-score PM (A) and C1 vs C2 primary mesothelioma cultures (B). The interquartile range method was applied to detect outliers. Mann-Whitney test. Error bars indicate SEM.
